# Data-driven collaborative QUality improvement in Cardiac Rehabilitation (QUICR) to increase program completion: protocol for a cluster randomized controlled trial

**DOI:** 10.1186/s12872-024-03971-3

**Published:** 2024-06-14

**Authors:** Dion Candelaria, Julie Redfern, Adrienne O’Neil, David Brieger, Robyn A Clark, Tom Briffa, Adrian Bauman, Karice Hyun, Michelle Cunich, Gemma A Figtree, Susie Cartledge, Robyn Gallagher

**Affiliations:** 1 Faculty of Medicine and Health, Susan Wakil School of Nursing and Midwifery, The University of Sydney, Sydney, NSW Australia; 2 Faculty of Medicine and Health, School of Health Sciences, The University of Sydney, Sydney, NSW Australia; 3 IMPACT - the Institute for Mental and Physical Health and Clinical Translation, Food & Mood Centre, School of Medicine, Deakin University, Barwon Health, Geelong, VIC Australia; 4 Faculty of Medicine and Health, Sydney Medical School, The University of Sydney, Sydney, NSW Australia; 5 Cardiology Department, Concord Hospital, ANZAC Research Institute, Sydney, NSW Australia; 6 Caring Futures Institute, Flinders University, Adelaide, SA Australia; 7 School of Population and Global Health, The University of Western Australia, Perth, WA Australia; 8 Faculty of Medicine and Health, School of Public Health, The University of Sydney, Sydney, NSW Australia; 9 Faculty of Medicine and Health, Central Clinical School, Charles Perkins Centre, The University of Sydney, Boden Initiative, Sydney, NSW Australia; 10 Sydney Local Health District, Camperdown, NSW Australia; 11 School of Public Health and Preventive Medicine, Monash University, Melbourne, VIC Australia

**Keywords:** Coronary heart disease, Cardiac rehabilitation, Data-driven, Quality improvement, Secondary prevention, Randomized controlled trial, Completion, Participation

## Abstract

**Background:**

Coronary heart disease (CHD) is the leading cause of deaths and disability worldwide. Cardiac rehabilitation (CR) effectively reduces the risk of future cardiac events and is strongly recommended in international clinical guidelines. However, CR program quality is highly variable with divergent data systems, which, when combined, potentially contribute to persistently low completion rates. The QUality Improvement in Cardiac Rehabilitation (QUICR) trial aims to determine whether a data-driven collaborative quality improvement intervention delivered at the program level over 12 months: (1) increases CR program completion in eligible patients with CHD (primary outcome), (2) reduces hospital admissions, emergency department presentations and deaths, and costs, (3) improves the proportion of patients receiving guideline-indicated CR according to national and international benchmarks, and (4) is feasible and sustainable for CR staff to implement routinely.

**Methods:**

QUICR is a multi-centre, type-2, hybrid effectiveness-implementation cluster-randomized controlled trial (cRCT) with 12-month follow-up. Eligible CR programs (*n* = 40) and the individual patient data within them (*n* ~ 2,000) recruited from two Australian states (New South Wales and Victoria) are randomized 1:1 to the intervention (collaborative quality improvement intervention that uses data to identify and manage gaps in care) or control (usual care with data collection only). This sample size is required to achieve 80% power to detect a difference in completion rate of 22%. Outcomes will be assessed using intention-to-treat principles. Mixed-effects linear and logistic regression models accounting for clusters within allocated groupings will be applied to analyse primary and secondary outcomes.

**Discussion:**

Addressing poor participation in CR by patients with CHD has been a longstanding challenge that needs innovative strategies to change the status-quo. This trial will harness the collaborative power of CR programs working simultaneously on common problem areas and using local data to drive performance. The use of data linkage for collection of outcomes offers an efficient way to evaluate this intervention and support the improvement of health service delivery.

**Ethics:**

Primary ethical approval was obtained from the Northern Sydney Local Health District Human Research Ethics Committee (2023/ETH01093), along with site-specific governance approvals.

**Trial registration:**

Australian New Zealand Clinical Trials Registry (ANZCTR) ACTRN12623001239651 (30/11/2023) (https://anzctr.org.au/Trial/Registration/TrialReview.aspx?id=386540&isReview=true).

## Background

Cardiovascular disease (CVD) is the leading cause of death globally, with coronary heart disease (CHD) accounting for nearly one-fifth of total deaths [1]. About 10% of people who survive a myocardial infarction experience another acute cardiac event within the first year [2], most of which are largely preventable [3, 4]. Comprehensive cardiac rehabilitation (CR) including structured exercise, psychosocial care, and timely patient education focussed on support for a healthy lifestyle and medication adherence is a cornerstone of care for patients with CHD [5, 6]. Patients who complete CR reduce their risk of repeat myocardial infarction (RR 0.72, 95% CI 0.55–0.93) and all-cause hospital admissions (RR 0.58, 95% CI 0.43–0.77) [7] accompanied by clinically meaningful improvements in functional capacity and health-related quality of life (HRQL) [7, 8]. Despite these benefits and strong recommendation of CR for the secondary prevention of CHD in international guidelines (Class 1 A evidence) [9], program participation rates continue to be suboptimal [10–12]. 

Poor attendance and completion of CR programs mean that many eligible patients fail to achieve the potential benefits of these programs. For instance, studies have demonstrated a dose-response benefit between exercise sessions attended and reductions in all-cause mortality [13] and risk of major adverse cardiovascular events (MACE) [14]. To achieve these benefits, it is recommended that CR programs offer a sufficient dose of exercise and efforts should focus on enabling patients to adhere to all prescribed sessions. Despite this, CR participation rates remain poor worldwide, with average rates ranging from 26.9% [10] to 48% [11]. Barriers to CR participation have been identified and include a combination of factors, both organisational (such as lack of adequate space and equipment) [15], and patient (such as not prioritising CR attendance amongst other life demands) [16]. There is growing evidence that suggests the quality of the CR program likely influences patient participation and thus outcomes [17]. National audits using quality indicators, performance measures, and key performance indicators (KPIs) developed by international peak bodies provide a means to evaluate the implementation of guideline-recommended therapies [18–21]. For instance, an audit of 170 programs in the United Kingdom found only 30.6% were considered high-quality according to their pre-determined minimum standards for CR (defined as having multidisciplinary team, offered to priority patient groups, and of adequate duration) [22]. In this audit, 5.3% does not meet any of the minimum standards and thus unlikely to facilitate patient participation or optimise outcomes [22]. Improving CR program quality may therefore be one of the keys to addressing low patient participation and completion.

One way to improve patient participation and completion of CR is by drawing on a Collaborative Quality Improvement Methodology, where various stakeholders work together toward a common goal of improving performance using a defined set of quality measures [23]. This collaborative approach enables rapid and sustainable changes in service delivery and, thus, has the potential to leverage the collective power of CR programs working simultaneously on shared problems and using data to drive performance [23]. Collaborative quality improvement has been successfully used in general practice [24], as well as in the management of asthma [25], and chronic heart failure [26], but to our knowledge, has not been tested for the optimal delivery of CR. Although a strategy of using purposeful and planned quality improvement initiatives demonstrated significant increase in patient attendance and completion in CR [27], this study was single-centre and non-randomized. Therefore, more evidence on the benefits of these initiatives is needed using robust research designs.

The Quality Improvement in Cardiac Rehabilitation (QUICR) trial aims to determine whether the implementation of a 12-month data-driven collaborative approach, relative to usual care:

1. increases CR program completion of patients with CHD (primary outcome),
2. reduces unplanned hospital admissions, emergency department presentations and deaths, and associated costs, and.
3. improves the proportion of patients receiving guideline-indicated CR according to national and international benchmarks, and

Furthermore, QUICR will include a process evaluation that runs parallel to the trial. This process evaluation will investigate the implementation of the collaborative approach and explore whether it is feasible and sustainable for CR staff to implement routinely, and identify enablers and barriers to implementation.

## Methods

This trial protocol follows the Standard Protocol Items: Recommendations for Interventional Trials (SPIRIT) checklist [28] and QUICR will be conducted in accordance with the Consolidated Standards of Reporting Trials (CONSORT) extension for cRCTs [29]. 

### Study Design

**Fig. 1:**
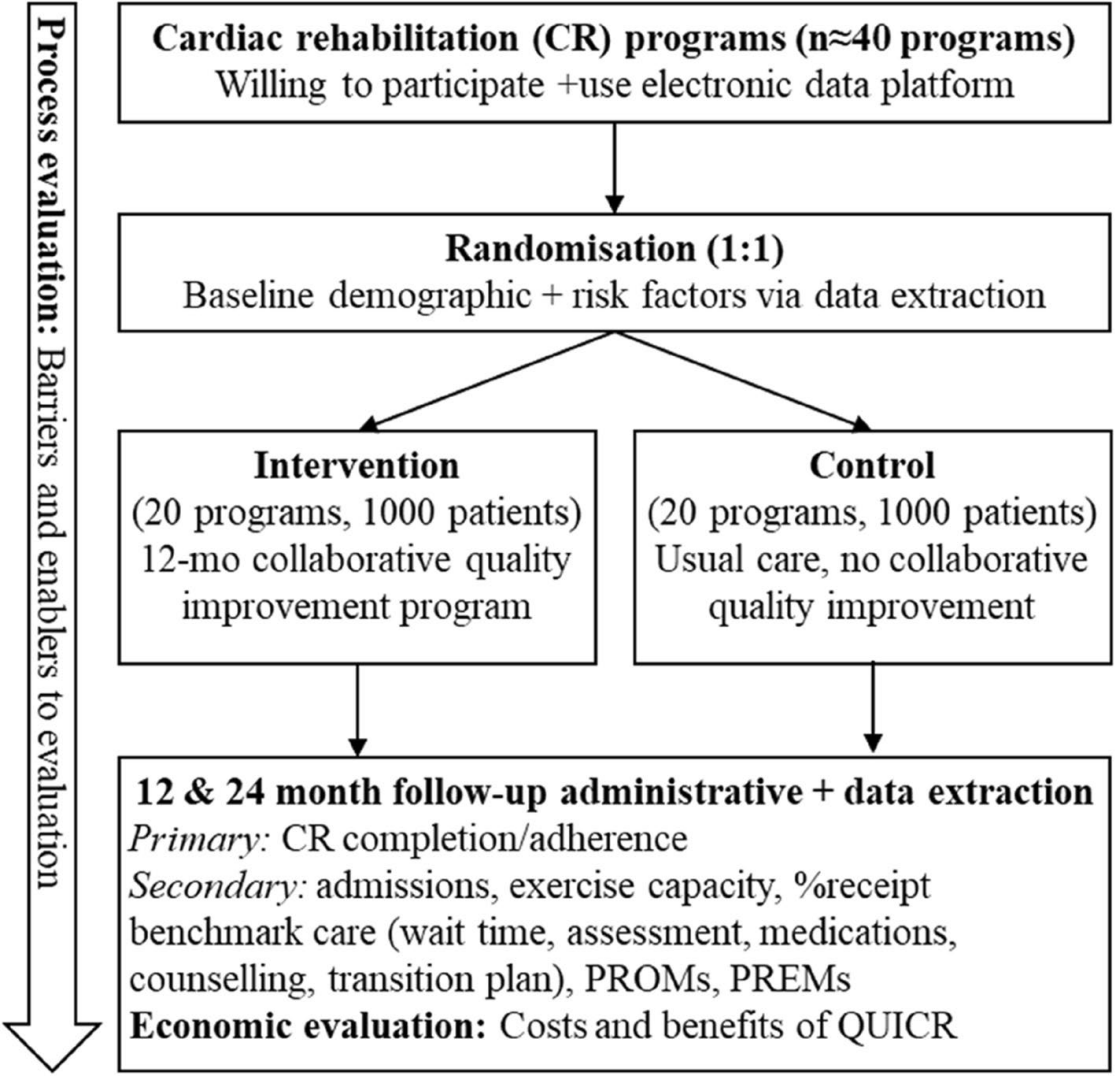
The QUICR Trial flow diagram

A type-2 hybrid effectiveness-implementation study [24], which uses a 2-arm, multi-centre, cluster-randomized controlled trial (cRCT) design will be conducted over 12 months (Fig. 1). The cRCT design enables accurate confirmatory causal inference [30]. The type-2 hybrid effectiveness-implementation study design, which has a dual focus on effectiveness and implementation outcomes, allows for simultaneous evaluation of the implementation intervention/strategy during an effectiveness trial [31]. 

### Study Setting/Recruitment

CR programs across two Australian states (New South Wales and Victoria) will be identified through our existing professional networks, societies, and available CR directories [32]. The research team will approach potential CR program leaders and provide information about the trial via email and/or phone call and during state-based in-person/online CR events. CR program leaders will be invited to ask questions and discuss any potential barriers to participation. Research team members will coordinate expressions of interest to participate, confirm eligibility, and commence formal recruitment processes.

### Program Eligibility Criteria

CR programs will be eligible to participate if they:

- enrol at least 70 patients eligible for CR per year,
- have internet-enabled computers, and.
- have staff with access to and proficiency in using the REDCap electronic collection data platform.

CR programs will be excluded if they are:

- unwilling to provide written agreement to participate in the quality improvement intervention or.
- already participating in a structured research project that entails changes to their usual CR program delivery.

The patient cohort for QUICR will comprise a dataset of all consecutive patients enrolled in participating CR programs who are ≥ 18 years old with a documented diagnosis of CHD in the medical record.

### Study outcomes

Table 1 outlines the QUICR KPIs. The primary outcome is CR program completion, defined as participation in ≥ 80% of sessions of the usual 6-8-week program. Secondary outcomes include: (1) unplanned hospital admissions, emergency department presentations and deaths, and associated costs via analysis of linked administrative data, (2) proportion of patients receiving guideline-indicated CR according to national and international benchmarks (e.g. assessment of risk factors, exercise capacity, medication adherence, and HRQL at entry and completion, and documented discharge transition plan), and (3) enablers and barriers to implementation of the QUICR intervention.

**Table 1:** QUICR Key Performance Indicators

1. Program completion
2. Wait time
3. Exercise capacity
A. Proportion of patients assessed for exercise capacity scores at initial assessment
B. Proportion of patients assessed for exercise capacity scores at completion assessment
C. Proportion of patients achieving improvement in exercise capacity
4. Low-density lipoprotein cholesterol (LDL-C)
A. Proportion of patients checked for LDL-C
5. Medication adherence
A. Proportion of patients assessed for medication adherence at initial assessment
B. Proportion of patients currently adhering to medication regimen at initial assessment
C. Proportion of patients assessed for medication adherence at completion assessment
D. Proportion of patients currently adhering to medication regimen at completion assessment
E. Change in proportion of patients currently adhering to medication regimen
6. Smoking
A. Proportion of patients assessed for smoking at initial assessment
B. Proportion of patients currently smoking offered/referred for smoking cessation counselling
C. Proportion of patients assessed for smoking at completion assessment
D. Proportion of patients currently smoking
E. Change in proportion of patients currently smoking
7. Depression
A. Proportion of patients screened for depression at initial assessment
B. Proportion of patients positive for depression offered/referred for counselling
C. Proportion of patients screened for depression at completion assessment
D. Proportion of patients positive for depression at completion assessment
E. Change in proportion of patients positive for depression
8. Health-related quality of life (HRQL) using EQ-5D-5 L
A. Proportion of patients assessed for HRQL at initial assessment
B. Proportion of patients assessed for HRQL at completion assessment
C. Proportion of patients achieving improvement in HRQL
9. Ongoing management plan
A. Proportion of patients with documented ongoing management plan for patient and GP
10. Patient experience survey
A. Proportion of patients who completed the patient experience survey

### Randomization

Each CR program will be randomized 1:1 to intervention (QUICR) or control group, via a computer-generated sequence allocation using simple randomization. CR programs that closely interact with each other were treated as a single cluster to circumvent the likelihood of contamination. Allocation will be stratified 50:50 by location of the programs (urban and regional areas) using SAS v9.4.

#### Blinding and allocation concealment

An independent statistician will generate the randomisation schedule so that the trial statistician remains blinded to group allocation for analysis. Given the design and nature of the quality improvement intervention where CR program staff enter data and use these to improve care, it is not possible to conceal the group allocation from the program staff themselves or the research team delivering the intervention. However, where possible, data collection is conducted blinded to treatment allocation (e.g. data linkage) as well as the statistical analyses.

### Intervention and control groups

#### Intervention Group

CR programs allocated to the intervention arm will participate in a multicomponent, data-driven quality improvement intervention (Table 2). The intervention is designed to use local CR data to drive small, progressive changes using Plan-Do-Study-Act (PDSA) cycles [33] aligned to pre-determined KPIs (Table 1). These indicators have been selected using national and international benchmarks of quality [18–21] and were developed and approved through an iterative process involving the trial investigators, CR clinicians, quality improvement experts, and consumers. The intervention will be guided by the Model for Improvement [34] using streamlined data entry and regular visual reports to programs via purpose-built databases using software systems REDCap and Microsoft PowerBI and underpinned by principles of collaboration and support. CR program leaders will collaborate in an ongoing way over 12 months supported and facilitated by the research team.

**Table 2:** Components of the QUICR collaborative quality improvement intervention

**Fig. 2:**
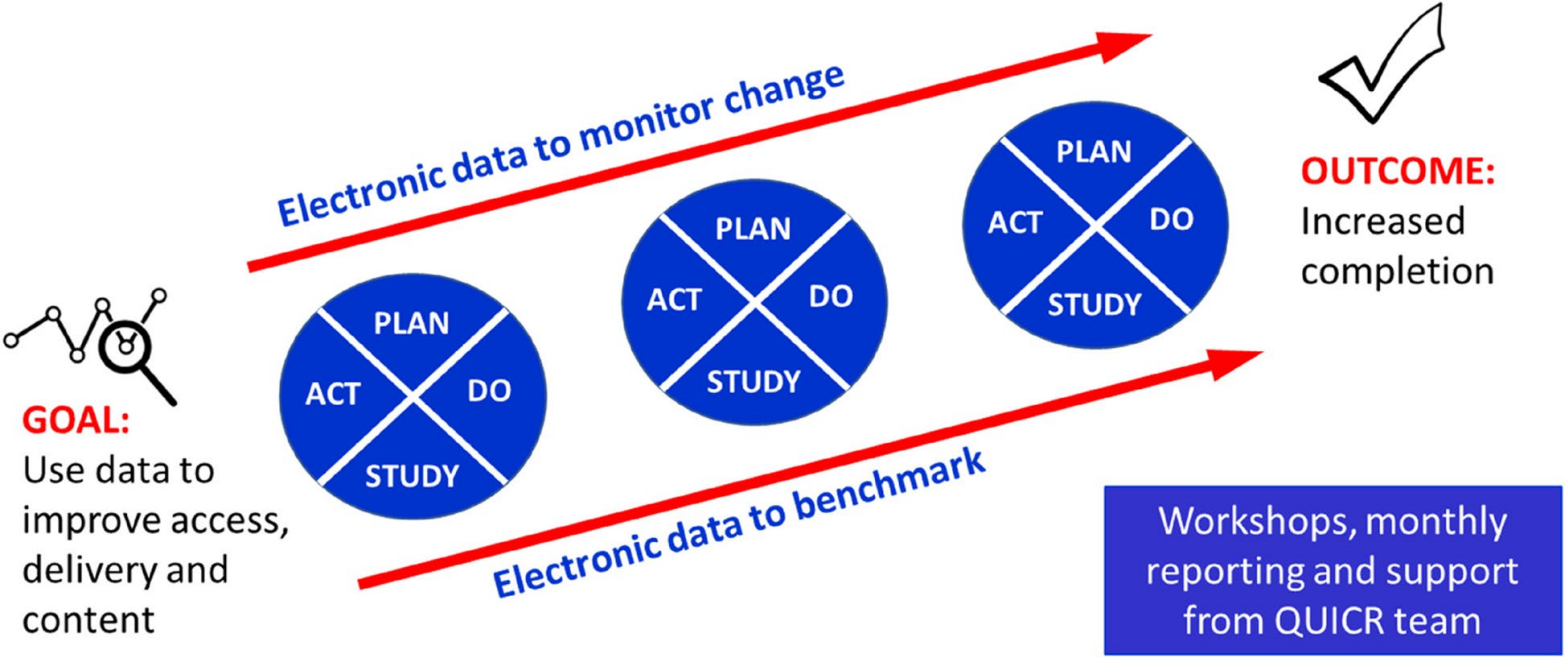
QUICR PDSA Diagram

1. **QUICR Dashboard and Monthly Reports**
Patient data entered into REDCap will be automatically extracted into a specially developed QUICR Dashboard to generate visual summaries of KPIs assessed and benchmarked against other participating programs anonymously. These monthly reports will provide CR program leaders accurate updates on their program’s performance in relation to KPIs over time.
2. **Collaborative Quality Improvement**
Regular group-based workshops (*n* = 4) (1 in-person and 3 online) will be held to support CR staff in developing quality improvement skills using the Model for Improvement and including PDSA cycles. As part of the collaborative approach, CR program representatives will be assisted to use their local data to identify gaps in best-practice care and initiate program-level changes that can be implemented and tested iteratively over time (Fig. 2). The initial group-based workshop will be six hours long and in-person for all programs in the intervention arm and occur within three months of beginning data collection to ensure reports are available. Workshop dates will be coordinated to minimise work disruption and the research team will support CR program representatives’ travel costs. Subsequent workshops will be three hours long, online, and scheduled every 2–3 months. Data reports for individual CR programs and collated overall performance of the collaborative will be discussed during the workshops and used to identify common areas for improvement. CR program representatives will work together in groups to identify and test potential solutions to shared problems. This collaborative approach will promote mutual learning among CR programs in addressing common issues. Research team members with expertise, skills, and prior experiences in collaborative quality improvement will facilitate all workshops.
3. **Facilitation and Support**
All participating programs in the intervention group will be supported by the research team for all aspects of the trial and the intervention. This team includes personnel trained in delivery of collaborative quality improvement to facilitate interpretation of monthly reports of analysed and visualised data, discuss quality improvement progress and objective feedback on PDSA cycle statements. All participating program staff will also have access to a secure online QUICR trial folder within a SharePoint site hosted by The University of Sydney that will house a suite of QUICR resources to ensure that CR programs have the learning resources they need and support channels to the research team to access in their own time. These resources will include the QUICR handbook, video training for data entry via REDCap and interpreting monthly reports, PDSA cycle worksheets, and frequently asked questions.

#### Control Group

CR programs allocated in the control arm are to provide usual care to their patients. For the purpose of measuring change in primary and secondary outcomes of intervention sites relative to control sites, staff working in CR at control sites are required to enter patient data into the purpose-built REDCap data collection platform. They will not have access to any of the abovementioned QUICR resources. These materials will be provided to the control group following trial completion.

### Data Collection and Management

All data will be collected at baseline, 12, and 24 months. The primary endpoint is 12 months. Individual patient-level data will be collected by CR program staff as part of routine care. De-identified demographic and CR patient outcomes data including costs will be entered into a purpose-built login-protected REDCap data collection platform hosted by the University of Sydney [35]. The master coding sheet with full patient identifiers will remain at each CR site’s server and will be the responsibility of the data custodian nominated at each program. Coding is crucial for this trial to enable access to and analyse administrative data for linkage. Data extracted to the QUICR Dashboard will be aggregated and automatically converted to visual form and no individual data point will be traceable. The QUICR Dashboard will also be login-protected, to which only a select number of the research team will have access.

Unplanned hospital admissions, emergency department presentations, and deaths will be collected via state-based linked administrative data. Probabilistic matching will be used to link records and the estimated proportion of invalid and missed links using data linkage is expected to be very low [36]. 

### Sample size

A sample of 40 CR programs (20/arm) with 33 patients with CHD in each program (660 patients/arm), will achieve 80% power to detect an increase in the CR completion rate of 22% from 59%. The control proportion estimated is 59% from our Australian National Cardiac Rehabilitation Quality Benchmark study (*n* = 2,436; 39 programs) [37]. The estimated change in completion rate is based on US data (*n* = 1103) reporting 25% increase in completions from quality improvement interventions [27]. We anticipate a slightly reduced effect as we are not using their small financial incentives. The intraclass correlation is 0.29, based on a cross-sectional study of cardiac rehabilitation [27]. The significance level of the test is 0.05.

### Statistical analyses

A study statistician blinded to the group allocation will lead all analyses according to a prespecified statistical analysis plan following intention-to-treat principles and consider CR program clustering. Descriptive statistics will be calculated and used to characterise patients, programs and KPIs. Mixed-effects linear and logistic regression models will be used for continuous and binary outcomes, respectively, accounting for clusters within the trial. Mixed-effects regression analysis adjusting for imbalances in patients at baseline will be conducted as sensitivity analysis. Prespecified subgroup analyses will be performed for gender, urban/regional CR sites, program size and duration, and private, public and community-funded programs. A detailed analysis plan will be developed and signed off prior to unblinding.

### Ethics

This trial will adhere to the National Health and Medical Research Council (NHMRC) ethical guidelines for human research [38] and the Ottawa Statement on the Ethical Design and Conduct of Cluster Randomized Trials [39]. Ethical approval has been obtained from the Northern Sydney Local Health District Human Research Ethics Committee (HREC) (2023/ETH01093). A waiver of patient consent has been granted because requiring individual consent introduces potential selection bias. Furthermore, patients in this trial will not be contacted for follow up and data linkage outcomes will be ascertained via data extraction from a reliable clinical software following robust governance processes overseen by national and State-based linkage units. This trial is registered with the Australian New Zealand Clinical Trials Registry (ANZCTR) (ACTRN12623001239651, Registered 30th November 2023) (anzctr.org.au). Any modifications to the trial protocol will be reported to the relevant HREC and ANZCTR.

### Economic evaluation of QUICR compared to Usual Care

Pertinent healthcare and other costs incurred in delivering the QUICR intervention will be identified by the research team and counted and valued using widely recognised methods [40, 41]. Key cost items include workforce, infrastructure and technology, equipment, and consumables; travel costs related to receiving CR and labour productivity costs to patients; and patient inpatient hospital admissions, emergency department presentations (and their respective stay duration and costs) during the intervention period. A specially built cost database will be developed by the research team to document the type of resources, quantity of each, and the value of these items. In addition, the trial will evaluate information on changes in healthcare utilisation and costs (hospital admissions and emergency department presentations) through data-linkage to State health administrative data. All these cost data along with effectiveness data such as CR completion, HRQL (EQ-5D-5 L) at program completion and reduction in unplanned hospital admissions will enable quantification of a broad range of costs and health outcomes for patients in the QUICR intervention and those in usual care at each site (and in total) as well as incorporating them in an assessment of cost-effectiveness.

### Process evaluation

A process evaluation for QUICR will be conducted parallel to the cRCT (Fig. 1). We will follow the process evaluations for cluster-randomised trials of complex interventions framework by Grant (2013), and unpack processes involving clusters (recruitment, delivery, responses) and individuals including CR staff and patients, as well as maintenance, effectiveness, and unintended consequences [42]. We will develop a logic model [43] to evaluate the implementation of the QUICR trial in terms of fidelity [44], processes and mechanisms, contexts [45], and theory [46] that contribute to causal pathways to intended/observed impacts. We will also identify barriers and enablers to implementation of the quality improvement intervention using a mixed methods approach.

We will collect quantitative and qualitative data throughout the QUICR intervention period using four sources:

1. *Pre–post surveys of CR staff knowledge, beliefs, and attitudes* towards collaborative quality improvement that will examine the uptake, feasibility, acceptability, utility, and sustainability of QUICR;
2. *Learning workshop attendance and quality improvement engagement records* to understand response to the QUICR intervention;
3. *PDSA cycle completion, quality, and application into programs* that indicate how the quality improvement is implemented and maintained;
4. *Focus group interviews of CR program staff representatives with detailed field notes* (*n* ≈ 20) at the start and end of the QUICR intervention. Focus group interviews at the start will assess the requisites for participating in QUICR including motivation, time commitment, staff skills and capacity, as well as learning needs and expectations. Information gathered in this focus group interviews will guide the content and delivery of succeeding workshops. The end-of-trial focus group interview will explore overall experiences with the QUICR intervention package including perceived barriers and enablers to implementation, sustainability, and unintended consequences. Recommendations for future enhancements of the QUICR intervention will also be sought.

For the process evaluation, descriptive statistics will be used to summarise quantitative data for the process evaluation. Maximum variation purposive sampling will be sought for the focus group interview participants for age, sex, and profession and to ensure data richness and saturation of the qualitative data [47]. Thematic analysis will be conducted [48] and will use context-method-outcome configuration to understand contextual influences [49]. 

## Conclusion

To the authors’ knowledge, this is the first RCT of this scale to evaluate the effectiveness of a CR program-level data-driven collaborative quality improvement intervention in CR aimed at improving completion in patients with CHD. Increasing the number of CR programs that provide best practice care promotes improved CR completion and patient outcomes at scale. The use of linked administrative data will enable evaluation of QUICR as a potentially efficient strategy for improving important secondary endpoints including reductions in hospital admissions, emergency department presentations, and deaths. This trial will also produce a robust health economic analysis and a process evaluation, specifically identifying barriers and enablers to the implementation of QUICR needed for optimisation of resources and sustainment beyond the trial completion.

### Dissemination

At the conclusion of the trial, results will be presented to local, national, and international scientific conferences and manuscripts submitted to peer-reviewed international journals following a publication protocol.

## Data Availability

The datasets generated and/or analysed will not be publicly available as the Ethical Review Board approval was obtained for public sharing and presentation of data on a group-level only. However, individual-level data may be available from the corresponding author on reasonable request noting that this will require separate ethics approval for the dissemination and use of the data.

## Abbreviations

ANZCTR: Australian New Zealand Clinical Trials Registry
CHD: Coronary heart disease
CONSORT: Consolidated Standards of Reporting Trials
CR: Cardiac rehabilitation
cRCT: Cluster-randomized controlled trial
CVD: Cardiovascular disease
HREC: Human Research Ethics Committee
HRQL: Health-related quality of life
KPI: Key performance indicator
MACE: Major adverse cardiovascular events
NHMRC: National Health and Medical Research Council
PDSA: Plan-Do-Study-Act
QUICR: QUality Improvement in Cardiac Rehabilitation
SPIRIT: Standard Protocol Items: Recommendations for Interventional Trials
